# Supplementary material for: Real-time drone derived thermal imagery outperforms traditional survey methods for an arboreal forest mammal
Source: PLoS One. 2020 Nov 16;15(11):e0242204. doi: 10.1371/journal.pone.0242204 (PMC7668579; doi:10.1371/journal.pone.0242204)

**Supporting Material for the research article:**

Real-time drone derived thermal imagery outperforms traditional survey methods for an arboreal forest mammal

Ryan R. Witt^1, 2*^, Chad T. Beranek^1, 2, 3^, Lachlan G. Howell^1, 2^, Shelby A. Ryan^1, 2^, John Clulow^1, 2^, Neil R. Jordan^4, 5^, Bob Denholm^3^ & Adam Roff ^1, 3^

^1^School of Environmental and Life Sciences, University of Newcastle, Callaghan, NSW, Australia.

^2^FAUNA Research Alliance, Kahibah, NSW, Australia.

^3^Science Division, NSW Department of Planning, Industry and Environment, Newcastle, NSW, Australia.

^4^Centre for Ecosystem Science, School of BEES, University of New South Wales (UNSW Sydney), Sydney, NSW, Australia.

^5^Taronga Institute of Science and Learning, Taronga Conservation Society Australia, Taronga Western Plains Zoo, Dubbo, NSW, Australia.

*Corresponding Author

Email: [ryan.witt@newcastle.edu.au](mailto:ryan.witt@newcastle.edu.au) (RRW)

**S1 File. Sightability parameter *f̂* (0) calculation for estimating koala density.**

The software program DISTANCE (release: 7.3) was used to calculate *f̂* (0) for Port Stephens from pooled distance data of 96 perpendicular observations of koalas collected across 73 line-transects (200 m in length) surveyed between January and July 2020, 6 repeats per site, on the Tomaree Peninsula.

Three candidate models were fitted to the data (**S1 Table**), and the best model was selected by assessing the AIC, effective strip half-width ($\hat{\mu}$) in metres, and the associated coefficient of variation (cv of $\hat{\mu}$). The analysis on DISTANCE suggested that a uniform key function with a cosine adjustment was the best model and the *f̂* (0) from this model (**S1 Fig**) was used to calculate the density of koalas at each of the three sites in which koalas were detected by spotlighting in Witt *et al.* 2020.

| **S1 Table.** **Analysis summary of three detection function models applied to koala line transect data collected from 73 transects lines over 6 repeated surveys on the Tomaree Peninsula, Port Stephens, NSW, Australia.** | | | | | | | |
| --- | --- | --- | --- | --- | --- | --- | --- |
| Key Function | Adjustment type | ∆AIC | AIC | $\hat{\mu}$ (m) | cv$(\hat{\mu}$) | 95% CI for $\mu$ | *f̂* (0) |
| Uniform | Cosine | 0.27 | 678.80 | 23.0 | 0.0394 | (21.3, 24.9) | 0.043425 |
| Half-normal | Hermite polynomial | 1.67 | 680.21 | 21.8 | 0.0795 | (18.7, 25.6) | 0.045793 |
| Hazard-rate | Simple polynomial | 0.00 | 678.54 | 23.8 | 0.0976 | (19.6, 28.8) | 0.042109 |

**S1 Fig. Sighting histogram and fitted detection function for the uniform cosine model for koalas sighted along 73 line transects over 6 repeated surveys on the Tomaree Peninsula, Port Stephens, NSW, Australia.**


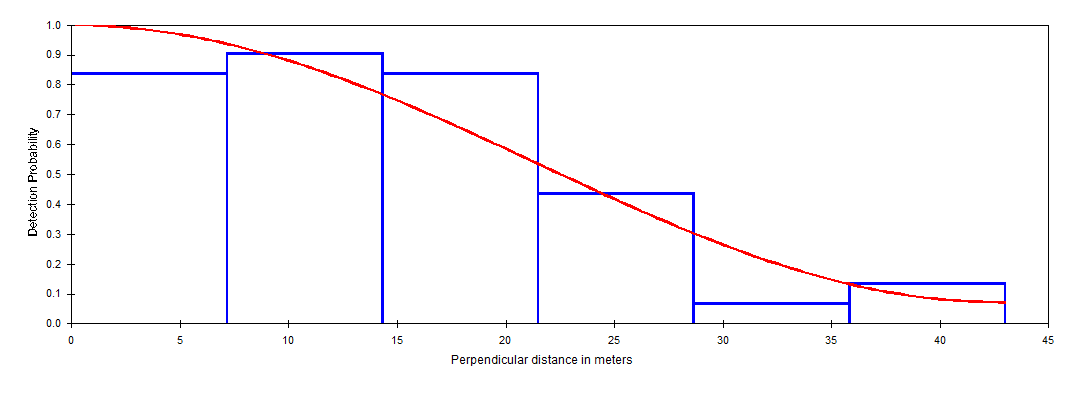

Supplement: S1 File — The software program DISTANCE (release: 7.3) was used to calculate f^(0) for Port Stephens from pooled distance data of 96 perpendicular observations of koalas collected across 73 line-transects (200 m in length) surveyed between January and July 2020, 6 repeats per site, on the Tomaree Peninsula. (DOCX) [file pone.0242204.s001.docx]
